# Effectiveness of an Enhanced Community Doula Intervention in a Safety Net Setting: A Randomized Controlled Trial

**DOI:** 10.1089/heq.2022.0200

**Published:** 2023-09-07

**Authors:** Julie Mottl-Santiago, Dmitry Dukhovny, Howard Cabral, Dona Rodrigues, Linda Spencer, Eduardo A. Valle, Emily Feinberg

**Affiliations:** ^1^ Department of Obstetrics and Gynecology, Boston Medical Center, Boston, Massachusetts, USA.; ^2^ Department of Pediatrics, Oregon Health & Science University, Portland, Oregon, USA.; ^3^ Department of Biostatistics, Boston University School of Public Health, Boston, Massachusetts, USA.; ^4^ Department of Community Health Sciences, Boston University School of Public Health, Boston, Massachusetts, USA.

**Keywords:** doula, maternal health, racial disparities, cesarean, breastfeeding, peer support

## Abstract

**Background:**

Racial inequities in maternal health outcomes, the result of systemic racism and social determinants of health, require maternity care systems to implement interventions that reduce disparities. One such approach may be support from a community doula, a health worker who provides emotional support, peer education, navigation, and advocacy for pregnant, birthing, and postpartum people who share similar racial identities, cultural backgrounds, and/or lived experiences. While community support during birth has a long tradition within communities of Black Indigenous and People of Color (BIPOC), the reframing of community doula support as a social intervention that reduces disparities in clinical outcomes is recent.

**Methods:**

We conducted a pragmatic randomized trial at an urban safety net hospital, comparing standard maternity care with standard care plus enhanced community doula support. We tested the effectiveness of a community doula program embedded in a safety net hospital in improving birth outcomes and explored the association between community doula support and health equity. Participants were nulliparous, insured by publicly funded health plans, and had lower risk pregnancies. The primary outcome was cesarean birth. Secondary outcomes included preterm birth and breastfeeding outcomes. Exploratory subgroup analysis was conducted by race–ethnicity.

**Results:**

Three hundred sixty-seven participants were included in the primary analysis. In the intent-to-treat analysis, outcomes were similar between groups. There was a trend toward increased breastfeeding initiation (*p*=0.08). There was a statistically nonsignificant 12% absolute reduction in cesarean birth and 11.5% increase in exclusive breastfeeding during delivery hospitalization among Black non-Hispanic participants.

**Discussion:**

While outcomes for the study sample were similar between randomization groups, health outcomes were improved for Black birthing people in cesarean and breastfeeding rates.

**Conclusion:**

This study demonstrates the need for larger studies of community doula support for Black birthing people. Clinicaltrials.gov ID: NCT02550730.

## Background

Racial inequities in maternal health outcomes are rooted in a long history of systemic racism, sexism, and classism in the United States.^1–3^ Black birthing people have higher rates of maternal morbidity, mortality, postpartum depression, and poor experience of maternity care and lower rates of breastfeeding than other racial groups.^4–11^ These outcomes are a result of centuries of macrolevel policies, institutional practices, and cultural norms that reinforce one another and continue to perpetuate social, economic, and political disadvantages for Black individuals and communities.^1,2^

Structural barriers to health experienced by Black people in the reproductive years include inequitable access to quality health care, housing, employment, education, community resources, and fair policing for themselves and their families.^12^ Within maternity care services, Black pregnant, birthing, and postpartum people report higher levels of obstetric violence, disrespect and dismissal, and withholding of information about their care.^13^

Black birth workers have always provided essential holistic health care to their communities in the United States.^14^ From the 17th to 19th centuries, enslaved Black midwives practiced the skills and traditions of African midwifery in caring for both other enslaved Black people and the White relatives of slave owners.^14^ In the Jim Crow era, “Grand” midwives in the South (elder Black community midwives) provided essential maternal and infant health care to Black communities.^15^

The American Medical Association's campaign to regulate the practice of medicine at the turn of the 20th century led to legal and regulatory limitations on the practice of community midwifery, including for Southern Black Grand midwives.^15^ At the same time, the rise of obstetrics as a field of medicine required training and practice opportunities for physicians, who were primarily White, upper class, and male.^15^ Childbirth was reframed as a medical event and moved rapidly into hospitals over the mid-20th century, eliminating community midwifery and support for Black birthing people.^15^

Community doulas have reclaimed this support role over the last few decades.^14,16,17^ As culturally congruent health workers, they share similar lived experiences and racial, cultural, and other intersectional identities with their client. Doulas provide physical and emotional support during pregnancy, childbirth, and the postpartum period. In the prenatal and postpartum periods, community doulas assist with navigation of health care and social services, provide peer education, and give social support. During labor, they provide continuous presence to promote physical comfort and support the birthing person's emotional needs.

Community doulas practice within a framework of birth justice,^16,17^ an aspect of reproductive justice, which names “the human right to maintain personal autonomy…” for Black birthing people.^17^ Doulas accomplish this through a variety of approaches.^16–19^ They navigate clients through resources essential for healthy social determinants of health (SDoH) such as housing, employment, nutritious food, and health care services that are less accessible compared with White pregnant and birthing people due to structural racism.^13,19^ They provide affirming and nonjudgmental support that may buffer the effects of relationship stressors, discrimination, and inadequate social support.^16,17^ In addition, they serve as advocates by amplifying the voice of the birthing person during labor and birth.^16,17^

A growing literature frames outcomes of community doula support as efficacious in reducing cesarean births,^18,20^ which may impact morbidity and mortality in both current and future pregnancies.^21^ Additionally, some studies show that doulas increase breastfeeding and improve the experience of maternity care.^18,20,22^ For low-income people and Black, Indigenous, People of Color (BIPOC), doulas provide a sense of physical and emotional safety, reduce stress related to experiences of discrimination in health care, and amplify the voice of the pregnant person.^16,17^

The effectiveness of community doula programs integrated into maternity care has not been studied. To understand the effectiveness of a doula program in improving health outcomes in a racially diverse low-income setting, a pragmatic, randomized controlled trial was conducted. The aims of the trial were to evaluate the effectiveness of doula support in reducing rates of cesarean and preterm births, as well as on breastfeeding outcomes. Additionally, the study aimed to explore the impact of the doula on health equity for Black birthing people, specifically focusing on cesarean birth outcomes.

## Materials and Methods

We conducted a parallel-group, pragmatic single-center trial with 1:1 randomization to assess the impact of community doula support on cesarean birth, preterm birth, and breastfeeding outcomes for nulliparous, lower-risk pregnant people with public insurance coverage. The study was conducted at an urban safety net hospital serving a racially and linguistically diverse population of ∼2700 births per year, of which 85% are publicly financed.

The institutional review board of the study site approved this study on April 29, 2015. Participants provided written informed consent. The study follows the Consolidated Standards of Reporting Trials (CONSORT) guidelines. We submitted our registration to Clinicaltrials.gov on June 23, 2015 (NCT02550730). We enrolled participants from August 2015 through November 2017. Data collection occurred through June 2018.

After completing the baseline survey, participants were randomized 1:1 in blocks of eight to either the Birth Sisters Best Beginnings for Babies (BBB) enhanced doula intervention or routine care. Computer-generated randomization was performed by an outside statistician and group allocation was placed in sequentially numbered opaque envelopes. The envelope was opened by the research assistant in front of the participant. The sequence was not revealed until enrollment was complete. Follow-up survey assessors were blinded to the allocation assignment of each participant.

### Participants

People pregnant with their first child, insured by Medicaid or other public insurance, and between 16 and 24 weeks of pregnancy were eligible. Research assistants assessed eligibility during routine ultrasound visits and obtained informed consent. Exclusion criteria were age <18 years, multiple gestation, known fetal anomaly, or high-risk pregnancy condition, defined by prenatal care attendance in the site's high-risk obstetric clinics.

This excluded people with substance use disorder, pre-existing diabetes, HIV infection, and other comorbidities requiring prenatal care from a maternal–fetal medicine specialist. Participants who developed gestational hypertension or diabetes after enrollment at 24 weeks of pregnancy were not excluded. Suicidal ideation during the baseline interview was also an exclusion criterion.

### Intervention

#### Birth Sisters BBB intervention

The intervention group received an enhanced model of Birth Sisters Program services starting at 24 weeks, known as the Birth Sisters BBB intervention. The Birth Sisters Program is one of the few hospital-based doula programs in the country. It has provided racially and ethnically diverse doula support to low-income pregnant and birthing people in an urban safety net hospital since 1999. The program and setting have been described in detail elsewhere.^23^

Briefly, Birth Sister services include between one and eight 2-h prenatal home visits determined by the client's preference; continuous support through labor and birth; and between one and four 2-h postpartum home visits through 6–8 weeks postpartum. Prenatal and postpartum activities include peer education, navigation of social and medical services, and social support. During labor, Birth Sisters provide physical and emotional comfort measures, as well as amplify the voice of the birthing person with the health care team.

Birth Sisters received training to maximize fidelity to the practice model before the study launch. Because this was an effectiveness study, strict adherence to the study protocol was not monitored. Instead, routine program management systems ensured fidelity. These include monthly group meetings and individual staff supervision at the beginning and close of each assignment with the Program Director, a midwife.

In addition to standard Birth Sister services, those in the intervention group received the enhancement of Medical Legal Partnership | Boston (MLPB) services to augment the ability of the Birth Sisters to address legally relevant SDoH. MLPB is a team of legal experts who integrate legal assistance into the medical setting so that low-income patients can meet legal needs that impact health. This enhancement aimed to maximize the role of the doula as an advocate around structural barriers to health and well-being for the individual client.

MLPB activities included training of Birth Sisters around SDoH resources, as well as serving as a consultant to the Birth Sister around individual participant needs. The training included an initial 3-h information session before participant enrollment on the basics of advocacy and a second 4-h training session on resources for unhoused clients. In the second year, a two-part training session, totaling 5 h, on resources around family law was provided.

In each year of the study, one to three Birth Sister group consultations during Birth Sister staff meetings were provided by MLPB lawyers to address individual case questions that might benefit the knowledge base for the entire doula staff. Additionally, each study participant assigned to the Birth Sisters Best Beginnings intervention was screened by the Birth Sister at 24 and 36 weeks for housing insecurity, food insecurity, and need for support around filling out the birth certificate.

When participants screened positive, the Birth Sister received a phone consultation with the MLPB lawyer for support around navigation resources. In the rare case that the participant required legal counsel, the Birth Sister was then able to refer the participant to MLPB for a *pro bono* formal consultation directly with the lawyer.

#### Routine care

Participants randomized to usual care had access to standard, interdisciplinary maternity care services at the safety net study site, including individual physician and midwifery care, group prenatal care that includes social support from other pregnant patients, childbirth education classes, social work support, in-patient lactation consultants, and 24-h interpreter services.

#### Outcomes

The primary outcome was the proportion of cesarean births. This outcome was selected for its potential effect on health equity since cesarean birth rates are highest among Black birthing people, correlated with increased rates of morbidity and mortality, and tied to obstetric racism. This outcome also aligned with prior doula research.

Secondary outcomes were preterm birth (<37 weeks 0 days of gestation), low birth weight (<2500 g at birth), breastfeeding initiation (any breast milk) and exclusivity (only breast milk) at delivery hospitalization, continuation of breastfeeding at the postpartum interview, Apgar scores <4 at 5 min, neonatal intensive care unit (NICU) admissions, and postpartum depression screening score defined by a score >9 on the Edinburgh Postnatal Depression Scale (EPDS) at the postpartum interview.

The definition of low Apgar score was selected based on the literature demonstrating correlation between an Apgar score of <4 and poor outcomes.^24^ Positive depression screen defined as a score >9 on the EPDS was chosen based on literature demonstrating high sensitivity and specificity at this cutoff.^25^

Obstetrical hemorrhage (quantitative blood loss >1000 mL) and hypertension (defined as at least two blood pressure readings >140/90 at least 4 h apart recorded in the medical record and/or in billing data) were added as outcome measures during the analysis phase due to increased focus nationally on reducing severe maternal morbidity and mortality.^26–28^

Data for outcomes measured at the delivery hospitalization were obtained from the electronic medical record. Breastfeeding continuation and postpartum depression outcomes were gathered at the postpartum interview.

*A priori* exploratory subgroup analyses were conducted by race to determine the differential impact by ethnic background and potential to reduce disparities, particularly for Black birthing people. Categories included Black non-Hispanic compared with all other races/ethnicities.

#### Sample size and power

Using a test of two independent proportions with a two-tailed alpha error of 0.05 and 80% power to detect a 10% absolute reduction in the rate of cesarean births, we required a sample size of 247 in each group of laboring participants. Assuming a loss to follow-up rate of 10%, a dropout rate of 5%, and scheduled cesarean rate (for medical indications) of 5%, we estimated a need for 297 participants in each group.

### Statistical analyses

Data were analyzed with SAS, version 9.4, using an intention-to-treat approach. A significance level of 0.05 was used. Analysis of the primary outcome included all participants who delivered at the study site, except for two postrandomization exclusions who were noted after enrollment to not meet inclusion criteria. Categorical variables were compared using either chi-square or Fisher's exact test, as appropriate. *p*-Values for the primary outcome and odds ratios with 95% confidence intervals for all outcomes were calculated.

For the outcomes of breastfeeding continuation and postpartum depression, logistic regression was used to control for prenatal breastfeeding intention and prenatal depression scores, respectively, as well as timing of the postpartum interview, as they were independent predictors of those outcomes.

Exploratory analyses stratified by race/ethnicity were preplanned and emphasized the estimation of subgroup-specific effects. We focused on exploring the historically large inequities in birth outcomes for Black birthing people. Hispanic, White, Asian, and Middle Eastern ethnicities were combined into a single category as the effect was similar between these groups.

We used the same statistical analysis approach for the intent-to-treat model described in the previous paragraph for the subgroup analysis. This exploratory analysis was not powered to detect statistically significant differences in outcomes.

To evaluate the treatment received, *post hoc* per-protocol analyses were conducted using logistic regression models. The issue of multiple testing was addressed by applying a reduced alpha level of 0.01 to assess statistical significance. The per-protocol intervention group consisted of all participants who delivered at the study site, who were assigned to the intervention group, and received at least one Birth Sister prenatal visit and labor support.

The control group consisted of all participants who delivered at the study site, who were assigned to the control group, and who did not receive any Birth Sister support. For breastfeeding continuation and postpartum depression outcomes, the per-protocol intervention group consisted of all participants who delivered at the study site, who were assigned to the intervention group, and received at least one Birth Sister prenatal visit, labor support, and at least one Birth Sister postpartum visit.

## Results

**FIG. 1.**
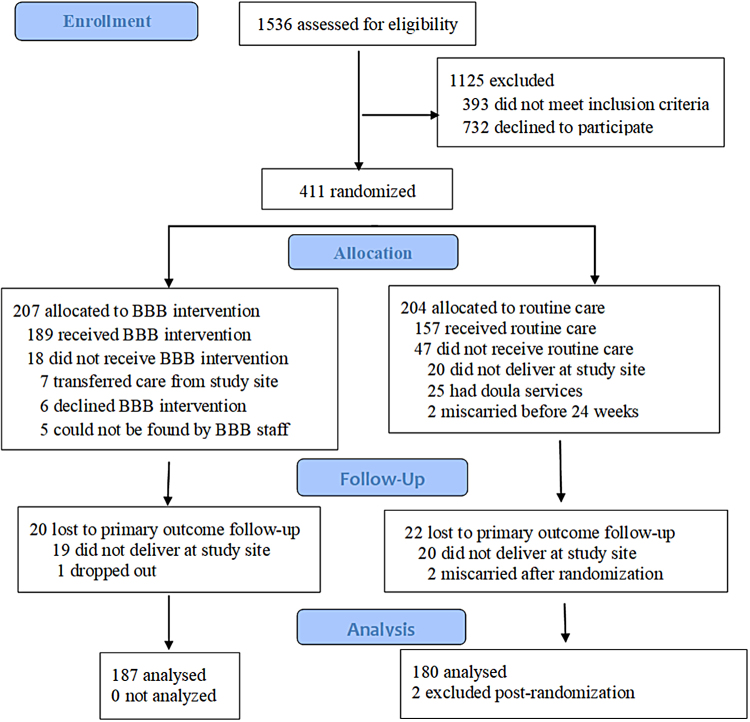
Flow diagram.

Figure 1 presents the trial's consort flow diagram. Four hundred eleven participants consented and were randomized. Of those, 367 (89%) remained in the analysis. Thirty-nine did not deliver at the study site, one dropped out, two miscarried before 24 weeks, and two were administratively withdrawn from the analysis due to subsequently noted exclusion conditions.

Three hundred thirteen of those who remained in the analysis for birth outcomes also completed a postpartum survey (85%). One hundred sixty of those participants completed the survey before 12 weeks postpartum. One hundred fifty-three completed the survey after 12 weeks of giving birth. The proportion of participants completing the survey before 12 weeks was similar between groups (51.6% in the intervention group compared with 52.6% in the control group).

We follow standard guidelines for reporting randomized trials.^29^ Our design achieved balanced groups, as noted in Table 1. Baseline characteristics for those who were lost to follow-up, dropped out, or excluded were also balanced between groups (Supplementary Table S1). All participants qualified for MassHealth, with an income of 200% of the federal poverty level. To measure differences in SDoH beyond income strata, we report baseline data on housing, food, and energy security,^30–32^ as well as social isolation.^33^

**Table 1.** Baseline Characteristics

Characteristic	Best beginnings (***n***=187)	Control (***n***=180)
Age in years, mean (SD)	25.4 (4.8)	25.5 (5.8)
Race/ethnicity, *n* (%)		
Hispanic	89 (47.6)	89 (49.4)
Non-Hispanic Black	67 (35.8)	63 (35.0)
Non-Hispanic White	13 (7.0)	12 (6.7)
Asian	10 (5.4)	6 (3.3)
Other	8 (4.3)	10 (5.6)
Natality,^a^ *n* (%)		
Non-U.S. born	142 (75.9)	135 (75.0)
U.S. born	45 (24.1)\	44 (24.4)
English fluency, *n* (%)		
I am fluent	93 (49.7)	97 (53.9)
I speak some English	58 (31.0)	58 (32.2)
I do not speak English	36 (19.3)	25 (13.9)
Prenatal care location, *n* (%)		
Hospital site	77 (41.2)	61 (33.9)
Community health center	110 (58.8)	119 (66.1)
Prenatal provider type, *n* (%)		
Midwife	77 (41.2)	83 (46.1)
OB	48 (25.7)	45 (25.0)
Family medicine	24 (12.8)	19 (10.6)
Other	8 (4.3)	4 (2.2)
Unsure	30 (16.0)	29 (16.1)
Group prenatal care	33 (17.7)	41 (22.8)
Food insecurity,^b^ *n* (%)	52 (25.1)	66 (32.3)
Housing insecurity,^c^ *n* (%)	44 (21.3)	48 (23.5)
Energy insecurity,^c^ *n* (%)	23 (11.1)	29 (14.2)
Social isolation,^d^ *n* (%)	22 (11.8)	24 (13.3)
Gestational age at enrollment, weeks (SD)	19.5 (1.53)	19.7 (1.50)

^a^ Control group had one “unknown,” not excluded from the main analysis.

^b^ Measured by the USDA Food Security Survey.^26^

^c^ Measured by the Children's Health Watch Questionnaire.^27,28^

^d^ Measured by a score of 6 or greater on the UCLA three-item Loneliness Scale.^29^

OB, obstetrician; SD, standard deviation.

### Intervention components and fidelity

Of 187 people included in the intervention group analysis, 172 (92%) participants received a prenatal visit, 142 (76%) received labor support, and 132 (71%) had a Birth Sister postpartum visit. Primary reasons for no labor support are as follows: 14 delivered at another hospital, 1 was lost to follow-up, 9 declined services after enrollment in the study, 4 delivered precipitously before the Birth Sister arrived, 12 gave birth without the Birth Sister being notified, and 5 were unknown.

The mean number of prenatal meeting hours was 5.3 (range 0–18.8), mean number of hours of labor support was 10.7 (range 0–25.5), and mean number of postpartum meeting hours was 3.1 (range 0–12.5). There was no difference in program fidelity by individual Birth Sisters. The mean caseload by Birth Sister was 13.2 (range 1–34) over the 28 months of enrollment. One hundred twenty-seven (91%) participants were matched with a doula who was racially congruent.

In the control group, 25 participants received a referral for the hospital's Birth Sisters Program through their clinical provider, although these participants did not receive the full BBB intervention, including MLP | Boston, since they were not enrolled in the intervention arm of the study. Thirteen received a Birth Sister prenatal visit, five had Birth Sister labor support, and eight had a Birth Sister postpartum visit. Typical program uptake is more reflective of the BBB intervention uptake described above.

### Intent-to-treat outcomes

As shown in Table 2, there were no significant difference between randomization groups in the primary outcome of cesarean birth (*p*=0.72). Overall breastfeeding initiation rates were high, but there was a trend (defined as a *p*-value of ≤0.1) toward increased breastfeeding initiation (*p*=0.08). Differences in Apgar scores <4 at 5 min were not statistically significant, but all fell in the control group. There were no differences in the other outcomes.

**Table 2.** Intent-to-treat Outcomes

Outcome	Best beginnings, ***n***=187	Control, ***n***=180	OR (95% CI)
Cesarean birth	53 (28.3)	54 (30.0)	0.92 (0.59–1.45)
Nulliparous term singleton vertex cesarean birth (*n*=329)	43 (26.1)	46 (28.1)	0.90 (0.56–1.47)
Obstetric hemorrhage	22 (11.8)	22 (12.2)	0.91 (0.49–1.70)
Gestational hypertension	22 (11.8)	20 (11.1)	1.07 (0.56–2.03)
Assisted vaginal birth	9 (4.8)	8 (4.4)	1.09 (0.41–2.88)
Epidural	119 (63.6)	114 (63.3)	0.99 (0.65–1.50)
Apgar score <4 at 5 min	0 (0.0)	3 (1.7)	0.14 (0.01–2.64)
Preterm birth	17 (9.1)	13 (7.2)	1.28 (0.61–2.73)
Low birth weight	16 (8.6)	14 (7.8)	1.11 (0.52–2.34)
Neonatal intensive care unit admission	24 (12.8)	22 (12.2)	1.06 (0.57–1.96)
Breastfeeding initiation	185 (98.9)	173 (96.1)	3.74 (0.77–18.26)
Breastfeeding exclusivity	78 (41.7)	82 (45.6)	0.86 (0.57–1.29)
Breastfeeding continuation^a^ (*n*=313)	94 (58.4)	89 (58.5)	1.03 (0.64–1.66)
Postpartum depression^b^ (*n*=313)	30 (18.6)	29 (19.1)	0.93 (0.50–1.72)

^a^ Adjusted by prenatal infant feeding intention and postpartum interview timing.

^b^ Adjusted by prenatal depression status and postpartum interview timing.

CI, confidence interval; OR, odds ratio.

### Exploratory analysis

Baseline characteristics by subgroups were balanced (Table 3). For Black non-Hispanic participants, there was an absolute reduction in cesarean births of 12.9% (28.4% vs. 41.3%). For nulliparous term singleton vertex (NTSV) births, the reduction was 14.5 absolute percentage points (25.9% vs. 40.4%). Breastfeeding exclusivity during the birth hospitalization increased from 33.3% to 44.8%.

**Table 3.** Exploratory Analysis for Non-Hispanic Black Race/Ethnicity

Outcome	Non-Hispanic Black		OR (95% CI)	Other^a^		OR (95% CI)
	**Best beginnings, *n*=67**	**Control, *n*=63**		**Best beginnings, *n*=120**	**Control, *n*=117**	
Cesarean birth	19 (28.4)	26 (41.3)	0.53 (0.28–1.20)	34 (28.3)	28 (23.9)	1.26 (0.70–2.25)
Nulliparous term singleton vertex cesarean birth (*n*=329)	15 (25.9)	23 (40.4)	0.52 (0.23–1.14)	28 (26.2)	23 (21.5)	1.29 (0.69–2.43)
Assisted vaginal birth	6 (9.0)	3 (4.8)	2.00 (4.80–8.36)	3 (2.5)	5 (4.3)	0.57 (0.13–2.46)
Obstetric hemorrhage	8 (11.9)	9 (14.3)	0.81 (0.29–2.26)	14 (11.7)	13 (11.1)	1.06 (0.47–2.36)
Gestational hypertension	11 (16.4)	13 (20.6)	0.76 (0.31–1.84)	11 (9.2)	7 (6.0)	1.59 (0.59–4.24)
Epidural in labor	44 (65.7)	39 (61.9)	1.18 (0.58–2.41)	75 (62.5)	75 (64.1)	0.93 (0.55–1.58)
Apgar score <4 at 5 min	0 (0.0)	1 (1.6)	0.48 (0.40–0.58)	0 (0.0)	2 (1.7)	0.49 (0.43–0.56)
Neonatal intensive care unit admission	9 (13.4)	10 (15.9)	0.82 (0.31–2.18)	15 (12.5)	12 (10.3)	1.25 (0.56–2.80)
Low birth weight	6 (9.0)	4 (6.4)	1.45 (0.39–5.40)	10 (8.3)	10 (8.5)	0.97 (0.39–2.43)
Preterm delivery	8 (11.9)	6 (9.5)	1.29 (0.42–3.95)	9 (7.5)	7 (6.0)	1.27 (0.46–3.54)
Breastfeeding initiation	67 (100)	61 (96.8)	2.10 (1.75–2.52)	118 (98.3)	112 (95.7)	2.63 (0.50–13.85)
Breastfeeding exclusivity	30 (44.8)	21 (33.3)	1.62 (0.80–3.30)	48 (40.0)	61 (52.1)	0.61 (0.37–1.02)
Breastfeeding continuation^b^ (*n*=313)	36 (64.3)	31 (57.4)	1.48 (0.65–3.37)	58 (55.2)	58 (59.2)	0.89 (0.49–1.62)
Postpartum depression^c^ (*n*=313)	15 (26.8)	13 (24.1)	1.49 (0.55–4.01)	15 (14.3)	16 (16.3)	1.01 (0.44–2.29)

^a^ Hispanic (*n*=178), non-Hispanic White (*n*=25), Asian (*n*=16), and Other (*n*=18).

^b^ Adjusted by prenatal infant feeding intention and postpartum interview timing.

^c^ Adjusted by prenatal depression status and postpartum interview timing.

For participants who were not Black non-Hispanic, the direction of the effect was reversed, with a 4.4% absolute increase in cesarean births overall and a similar increase of 4.7% for NTSV cesarean births. There was also a reversal of direction for exclusive breastfeeding, decreasing from 52.1% to 40.0%.

### Per-protocol analysis

In the per-protocol analysis, 138 of 187 (73.7%) participants in the intervention group and 156 of 180 (86.7%) in the control group were included (Table 4). There was no statistically significant difference in the primary outcome (*p*=0.39) or any of the secondary outcomes. Preterm birth, low birth weight, and NICU admissions were lower for the intervention group, which is a change in the direction of the effect from the intent-to-treat analysis.

**Table 4.** Per-protocol Analysis

Outcome	Best beginnings, ***n***=138	Control, ***n***=156	Adjusted OR (99% CI)
Cesarean birth^a^	37 (26.8)	49 (31.4)	0.79 (0.40–1.58)
Nulliparous term singleton vertex cesarean birth^a^ (*n*=268)	34 (26.6)	41 (29.3)	0.86 (0.41–1.78)
Assisted vaginal birth^b^	6 (4.4)	6 (3.9)	1.55 (0.31–7.88)
Obstetric hemorrhage^c^	15 (10.9)	21 (13.5)	0.83 (0.40–1.73)
Gestational hypertension^d^	14 (10.1)	17 (10.9)	1.00 (0.36–2.78)
Epidural^e^	96 (69.6)	99 (63.5)	1.34 (0.82–2.19)
Apgar score <4 at 5 min^f^	0 (0.0)	3 (1.9)	0.52 (0.46–0.61)
Low birth weight^g^	6 (4.4)	12 (7.7)	0.63 (0.16–2.45)
Preterm delivery^g^	9 (6.5)	13 (8.3)	0.80 (0.24–2.60)
Neonatal intensive care unit admission^h^	13 (9.4)	22 (14.1)	0.75 (0.23–2.45)
Breastfeeding initiation^e^	138 (100.0)	149 (95.5)	1.05 (1.00–1.09)
Breastfeeding exclusivity^i^	60 (43.5)	74 (47.4)	0.82 (0.44–1.51)
Breastfeeding continuation^j^ (*n*=252)	74 (61.7)	79 (59.9)	1.11 (0.52–2.40)
Postpartum depression^k^ (*n*=252)	21 (17.5)	21 (15.9)	0.92 (0.32–2.60)

^a^ Adjusted by age and prenatal care location.

^b^ Adjusted by age, race, and prenatal care location.

^c^ Adjusted by prenatal care location, race, and food security.

^d^ Adjusted by race, prenatal provider type, and natality.

^e^ Adjusted by English fluency.

^f^ Adjusted by no adjustment.

^g^ Adjusted by natality and housing security.

^h^ Adjusted by race, prenatal care location, preterm birth, low birth weight, and food security.

^i^ Adjusted by food security.

^j^ Adjusted by postpartum interview timing, prenatal feeding plan, natality, food security status, prenatal care location, race, and English fluency.

^k^ Adjusted by postpartum interview timing, prenatal depression screening, prenatal care location, and English fluency.

## Discussion

This effectiveness trial did not show significant differences in outcomes between randomization groups. The single-site intention-to-treat analysis, while maximizing the internal validity of the study, limits its generalizability to other settings. Additionally, nonadherence to the assigned treatment and loss to follow-up in pragmatic trials can make interpretation of the intent-to-treat analysis unclear.^34^

Our per-protocol analysis attempts to understand how participants would benefit from the intervention with full adherence to the randomized treatment.^34^ However, a per-protocol analysis loses the benefits of randomization in reducing bias.

We followed standard statistical analysis recommendations for per-protocol analyses by adjusting for confounders and specifying a more restrictive definition of significance.^34^ The larger magnitude of absolute reduction in cesarean births in the per-protocol compared with the intent-to-treat analysis (4.6% vs. 1.7%) and reversal of direction for preterm birth outcomes are more consistent with prior studies.^18,20^

The subgroup analysis by race suggests that the BBB intervention may improve cesarean and breastfeeding outcomes for Black non-Hispanic participants. These results are exploratory and were not powered to find statistically meaningful differences between groups. However, based on a scan of the literature in PubMed, this study is the first trial to examine the relationship between racially/culturally congruent doula support and maternal health outcomes.

The reduction in racial disparities for Black non-Hispanic people in this trial has important implications for advancing health equity. As noted previously, cesarean birth rates are higher in Black non-Hispanic populations, are correlated with higher rates of morbidity and mortality, and are related to obstetric racism. Breastfeeding rates are also lower for Black populations as a result of historical and current systemic racism.^7,35^

The important advantages of breastfeeding for the health of both infant and mother are well documented.^36^ The finding that community doulas may improve these rates is important for understanding the potential intergenerational impact of doulas on health and gives support to the call for doulas as an integral part of birthing care for Black people.^37^

Health equity, according to the CDC, is defined as “the state in which everyone has a fair and just opportunity to attain their highest level of health. Achieving this requires focused and ongoing societal efforts to address historical and contemporary injustices; overcome economic, social, and other obstacles to health and healthcare; and eliminate preventable health disparities.”^38^

The BBB intervention achieves this definition of health equity in three ways. First, it addresses historical injustices rooted in the systematic elimination of Black birth workers in the United States by recognizing the experience and expertise of Black birthing people and their communities. Second, the BBB intervention helps birthing people connect with resources that reduce economic and social barriers to health. Third, it reduces disparities in cesarean birth and breastfeeding outcomes between Black birthing people and those of other races/ethnicities.

We did not compare disparities in outcomes between Black and White groups due to the small number of White non-Hispanic participants. Nationally, White, non-Hispanic, nulliparous birthing people have a low-risk cesarean rate of 24.9% similar to the low-risk cesarean birth rate of 25.9% for Black non-Hispanic participants in the intervention group, down from 41.3% in the control group.^39^

The literature offers suggestions about why the use of community doulas may be an effective strategy to achieve health equity for Black birthing people. Community doulas ensure that racially and culturally congruent emotional, physical, and informational support is available for those most at risk for experiencing structural racism and bias in health care.^17,27,36,37^ Emotional support may particularly influence Black maternal health since anti-Black racism is pervasive and emotionally draining.

Qualitative studies of doulas serving Black people show that racial congruence and shared lived experience are valued by doula clients and promote a trusting relationship that mitigates the effects of racism in maternity care.^40,41^ Survey research also suggests that the presence of the doula improves the experience of respectful care for Black people,^42^ potentially reducing unnecessary cesarean births. This outcome is also important for reducing disparities in maternal morbidity since cesarean births have higher rates of hemorrhage and complications in both current and future pregnancies.^21^

It is unclear why the subgroup analyses showed worse outcomes in the other subgroups. Perhaps cesarean rates in the control group were low enough that further reduction would require a different type of intervention. Alternatively, since the exploratory analyses were not powered to find statistically significant differences in outcomes, the stratified analysis results could be due to chance.

### Limitations

The primary limitation of this study is that it enrolled 411 rather than the 494 participants needed to find the expected change in the primary outcome. Additionally, the 10% absolute reduction anticipated for the power calculation overestimated the magnitude of change. The exploratory study, by definition, was also not powered to find statistically meaningful differences between groups for the primary outcome of cesarean birth.

Additionally, obstetric racism in and of itself is a negative outcome and the narrow focus on the primary outcome of cesarean birth is a limitation for understanding the larger impact of doulas on health equity.^43,44^ Finally, the exclusion of higher risk pregnancies is also a limitation for fully understanding the impact of doulas on Black birthing people since they suffer a disproportionate burden of pre-existing diabetes and hypertension.

## Conclusions

This trial provides useful information for future research on the impact of doulas on advancing health equity. Future trials should enroll larger numbers of Black birthing people and anticipate smaller changes in cesarean birth. Qualitative and ethnographic research should also investigate why community doula support is particularly impactful for Black birthing people.

## Supplementary Material

Supplemental data

## Abbreviations Used

BBB: Best Beginnings for Babies
BIPOC: Black, Indigenous, and People of Color
CDC: Centers for Disease Control and Prevention
CI: confidence interval
MLP: Medical Legal Partnership
NICU: neonatal intensive care unit
NTSV: nulliparous term singleton vertex
OB: obstetrician
OR: odds ratio
SD: standard deviation
SDoH: social determinants of health
