# Supplementary material for: Effectiveness of an Enhanced Community Doula Intervention in a Safety Net Setting: A Randomized Controlled Trial
Source: Health Equity. 2023 Sep 7;7(1):466–76. doi: 10.1089/heq.2022.0200 (PMC10507922; doi:10.1089/heq.2022.0200)
Supplement: Supplemental data [file Suppl_TableS1.docx]

Supplement 1: Baseline characteristics of those who did not complete the study

| **Characteristic** | **BBB** | **Control** |
| --- | --- | --- |
| **Age in years, mean (SD)** | 23.1 yrs +/- 5.1 | 25.6 +/- 5.2 |
| **Race/ethnicity n (%)**  **Hispanic**  **Non-Hispanic Black**  **Non-Hispanic White**  **Asian**  **Other** | 14 (70.0)  6 (30)  0 (0.0)  0 (0.0)  0 (0.0) | 9 (37.5)  11 (45.8)  1 (4.2)  2 (8.3)  1 (4.2) |
| **Natality^*^ n (%)**  **Non-US born**  **US born** | 12 (60.0)  8 (40.0) | 12 (50.0)  12 (50.0) |
| **English fluency n (%)**  **I am fluent**  **I speak some English**  **I do not speak English** | 11 (55.0)  8 (40.0)  1 (5.0) | 13 (54.9)  6 (25.0)  5 (20.8) |
| **Prenatal care location n (%)**  **Hospital site**  **Community Health Center** | 0 (0,0)  20(100.0) | 0 (0.0)  24 (100.0) |
| **Prenatal provider type n (%)**  **Midwife**  **OB**  **Family Medicine**  **Other**  **Unsure** | 10 (50.0)  3 (15.0)  1 (5.0)  2 (10.0)  4 (20.0) | 11 (45.8)  4(16.7)  3 (12.5)  0 (0.0)  6 (25.0) |
| **Group prenatal care n(%)** | 5 (25.0) | 7 (29.2) |
| **Food insecurity^b^ n (%)** | 8 (40.0) | 133 (54.2) |
| **Housing insecurity^c^ n (%)** | 7 (35.0) | 9 (37.5) |
| **Energy insecurity^c^ n (%)** | 2 (10.0) | 4 (16.7) |
| **Social isolation^d^ n (%)** | 2 (10.0) | 6 (25.0) |
| **Gestational age at enrollment, weeks (SD)** | 20.6 +/- 2.3 | 19.0 +/- 2.2 |
